# Human pandemic K27-ST392 CTX-M-15 extended-spectrum β-lactamase-positive *Klebsiella pneumoniae*: A one health clone threatening companion animals

**DOI:** 10.1016/j.onehlt.2022.100414

**Published:** 2022-07-03

**Authors:** Luciano C.B.A. da Silva, Brenda Cardoso, Herrison Fontana, Fernanda Esposito, Silvia R.G. Cortopassi, Fábio P. Sellera, Nilton Lincopan

**Affiliations:** a Department of Surgery, School of Veterinary Medicine and Animal Science, University of São Paulo, São Paulo, Brazil; b School of Veterinary Medicine, Metropolitan University of Santos, Santos, Brazil; c One Health Brazilian Resistance Project (OneBR), São Paulo, Brazil; d Department of Microbiology, Institute of Biomedical Sciences, University of São Paulo, São Paulo, Brazil; e Department of Clinical Analysis, Faculty of Pharmaceutical Sciences, University of São Paulo, São Paulo, Brazil; f Department of Internal Medicine, School of Veterinary Medicine and Animal Science, University of São Paulo, São Paulo, Brazil

**Keywords:** Antimicrobial resistance, Critical-priority pathogen, Enterobacterales, One health, Veterinary medicine, Genomic surveillance

## Abstract

Extended spectrum β-lactamase (ESBL)-producing *Klebsiella pneumoniae* is a medically important pathogen that commonly causes human nosocomial infections. Since veterinary emergency and critical care services have also significantly progressed over the last decades, there are increasing reports of ESBL-producing *K. pneumoniae* causing hospital-associated infections in companion animals. We present microbiological and genomic analysis of a multidrug-resistant ESBL-positive *K. pneumoniae* (LCKp01) isolated from a fatal infection in a dog admitted to a veterinary intensive care unit. LCKp01 strain belonged to the sequence type ST392 and displays a KL27 (wzi-187) and O-locus 4 (O4). A broad resistome and presence of the *bla*_CTX-M-15_ ESBL gene were predicted. SNP-based phylogenomic analysis, using an international genome database, clustered LCKp01 (60–80 SNPs differences) with *K. pneumoniae* ST392 from human and animal infections, isolated at 4-year interval, whereas phylogeographical analysis confirmed successful expansion of ST392 as a global clone of One Health concern.

## Highlights

• A fatal infection by a multidrug-resistant *K. pneumoniae* in dog was investigated.
• Identification of human pandemic *K. pneumoniae* ST392/CTX-M-15 clone is highlighted.
• Phylogenomic analysis revealed clonal relatedness with nosocomial lineages.
• Phylogeographical analysis confirmed expansion of ST392 as global One Health clone.
• Dissemination of *K. pneumoniae* ST392 at the human-animal interface is discussed.

## Graphical abstract

**Figure:**
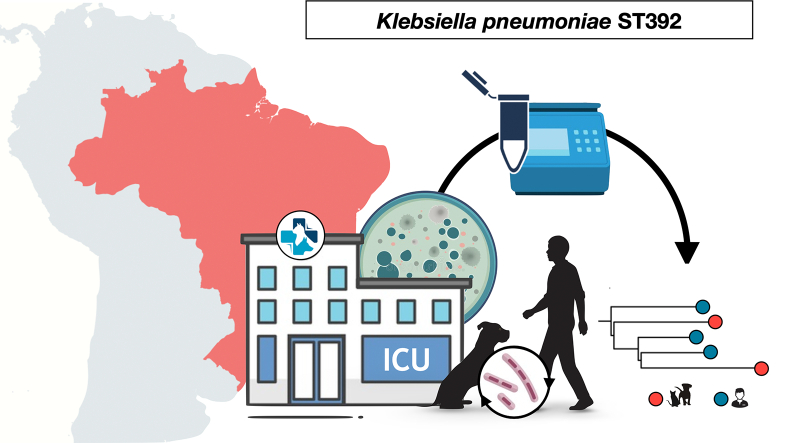

## 1 Introduction

*Klebsiella pneumoniae* is a clinically relevant pathogen frequently associated with antimicrobial resistance, being considered an important cause of community-acquired and nosocomial infections in humans [1]. In this regard, after the global spread of extended spectrum β-lactamase (ESBL)-producing *K. pneumoniae* causing human nosocomial infections, these bacteria are currently emerging as worrisome causes of infections in pets worldwide [2,3]. Due to their clinical and epidemiological importance, the World Health Organization (WHO) has recently classified ESBL-producing *K. pneumoniae* isolates as critical priority pathogens [4]. In view of the wide diversity of ESBL-positive *K. pneumoniae* being recovered from humans, animals, and the environment, the One Health approach has been encouraged for a better understanding of the clonal spread of these strains [5].

Analogously to human medicine, small animal patients hospitalized in intensive care units (ICUs) are mostly affected by life-threatening infections, including those caused by multidrug-resistant (MDR) pathogens [6]. Indeed, since veterinary emergency and critical care services have progressed meaningfully in recent times, increasing reports of ESBL-producing pathogens causing hospital-associated infections in companion animals could be expected [[6], [7], [8]]. Therefore, the use of invasive devices, antimicrobial prescribing practices, and increased hospital stay could be predisposing factors for the acquisition of ESBL-producing *K. pneumoniae* infections by pets [6,7], which poses a substantial challenge for veterinary clinicians, being also a One Health issue.

## 2 Materials and methods

In 2018, a 2-year-old mixed breed female dog was admitted to a veterinary ICU after gastrointestinal surgery procedures. Veterinary medical records revealed that the dog was subjected to two laparotomies ten days before ICU admission. In the first procedure, gastrostomy and enterostomy were performed for foreign body removal. During the postoperative period, the dog developed surgical wound dehiscence and a significant amount of intra-abdominal free fluid was observed during ultrasonography. On account of these complications, a second laparotomy procedure for enterectomy and anastomosis was performed. Following 3 days of the second procedure, the dog was referred to the ICU due to significant hemodynamic instability, abdominal pain, peritoneal fluid escaping from the abdominal cavity, decreased consciousness, and inappetence. A peritoneal fluid sample was collected and subjected to bacteriological culture and antimicrobial susceptibility testing. Antibiotic therapy with enrofloxacin and metronidazole was started, and support treatment was immediately performed, however, the animal died 36 h following ICU admission.

*K. pneumoniae* isolate (LCKp01) was recovered from the peritoneal fluid, being identified by BD Phoenix (BD Diagnostics, Sparks, MD). The *K. pneumoniae* strain LCKp01 exhibited a MDR profile to amoxicillin/clavulanic acid, ceftiofur (MIC >32 μg/ mL), ceftazidime, cefotaxime, cefepime, sulfamethoxazole/trimethoprim, enrofloxacin, ciprofloxacin, gentamicin, levofloxacin, nalidixic acid, and tetracycline. Otherwise, it remained susceptible to amikacin, ertapenem, imipenem, meropenem, cefoxitin, aztreonam, amikacin and fosfomycin as determined by disc diffusion and/or *E*-test methods [9]. Additionally, ESBL production was confirmed by using a double-disc synergy test, whereas PCR screening and direct sequencing identified the *bla*_CTX-M-15_ gene.

We performed whole-genome sequencing (WGS) of the *K. pneumoniae* LCKp01 using an Illumina MiSeq platform with 300-bp read lengths. Reads were trimmed using TrimGalore v0.6.5 (https://github.com/FelixKrueger/TrimGalore) and *de novo* assembled with Unicycler v.0.4.8 (https://github.com/rrwick/Unicycler). Annotation was automatically NCBI Prokaryotic Genome Annotation Pipeline (PGAP) v.3.2 (http://www.ncbi.nlm.nih.gov/genome/annotation_prok/). Multilocus sequence typing (MLST), capsular serotyping, wzi-locus identification, and point mutations were predicted using Kleborate v.2.1.0 (https://github.com/katholt/Kleborate). Antimicrobial resistance genes, plasmid Inc-type identification and plasmid MLST (pMLST) assignment were performed using tools from the Center for Genomic Epidemiology web server (http://genomicepidemiology.org).

To address the phylogenomic relatedness of LCKp01 from a global perspective, we analyzed 12.126 genomes of *K. pneumoniae* from the NCBI RefSeq database. We retrieved 66 genomes of *K. pneumoniae* ST392, and a maximum-likelihood tree based on SNP alignment was inferred with default parameters of CSI Phylogeny v.1.4 (https://cge.cbs.dtu.dk/services/CSIPhylogeny), using the complete chromosome of *K. pneumoniae* ST392 strain KPNIH31 (GenBank accession number: CP009876.1) as reference. Tree topology visualization and annotation were performed with iTol v.6 (https://itol.embl.de/). Genotypic typing of K (capsule) and O antigen (LPS) serotype prediction using wzi alleles, as well as ICEKp virulence associated locus were identified with Kleborate v.2.2.0 (https://github.com/katholt/Kleborate), whereas acquired antimicrobial resistance genes were identified using the Resfinder v.4.1 (https://bitbucket.org/genomicepidemiology/resfinder_db.git/src) database within Abricate (https://github.com/tseemann/abricate) software, using a minimum threshold of 90% for nucleotide identity and gene coverage.

## 3 Results and discussion

**Fig. 1:**
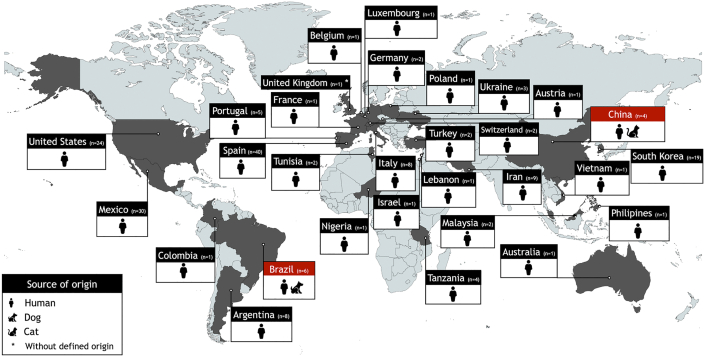
Worldwide distribution and sources of *Klebsiella pneumoniae* of ST392 circulating at the One Health interface. Data were retrieved from PubMed database *via* the National Center for Biotechnology Information (NCBI) interface and NCBI RefSeq database (http://www.ncbi.nlm.nih.gov/RefSeq/) (accessed on April 27, 2022). Duplicates (*i.e.,* the same *K. pneumoniae* ST392 strain in both databases) were excluded.

The genome size of LCKp01 was calculated at 5,782,901 bp, comprising 5701 total genes, 3rRNAs and80 tRNAs and 10 non-coding RNA, with 322× coverage. Multilocus sequence typing analysis showed that *K. pneumoniae* LCKp01 strain was assigned to ST392. Interestingly, this *K. pneumoniae* lineage has been observed so far in humans in Africa, Asia, Europe, Oceania, and in the Americas (Fig. 1); being frequently recovered from nosocomial infections with the production of CTX-M-type ESBLs and/or carbapenemases [10,11]. To the best of our knowledge, there is a single *K. pneumoniae* ST392 strain isolated from animals, and it was recovered from a cat suffering from urinary tract infection in China, in 2018.

There are few reports of *K. pneumoniae* ST392 in the literature; however, some studies suggest the emergence of ST392 isolates with the potential to become a lineage of clinical relevance [11,12]. A recent investigation focused on phenotypical and molecular assessment of the virulence potential of *K. pneumoniae* ST392 clinical isolates revealing that most of the strains were highly resistant to human sera and were also strong biofilm producers, showing strong levels of adhesion to the HT-29 epithelial intestinal cell line [11]. These phenotypic behaviors were associated to the presence of genes involved in serum resistance (*aroE* and *traT*) and adhesion (*pgaA*) [11]. Although, *in silico* analysis of *K. pneumoniae* LCKp01 revealed the presence of *aroE, traT,* and *pgaA* genes, the existence of these genes alone is not sufficient to infer that LCKp01 is a strong biofilm producer or highly resistant to serum. Therefore, *in vitro* serum resistance and biofilm assays must be investigated, in order to elucidate whether these genes could contribute with persistence of LCKp01 in medical devices and healthcare-associated infections in human and veterinary medicine [[13], [14], [15]].

**Fig. 2:**
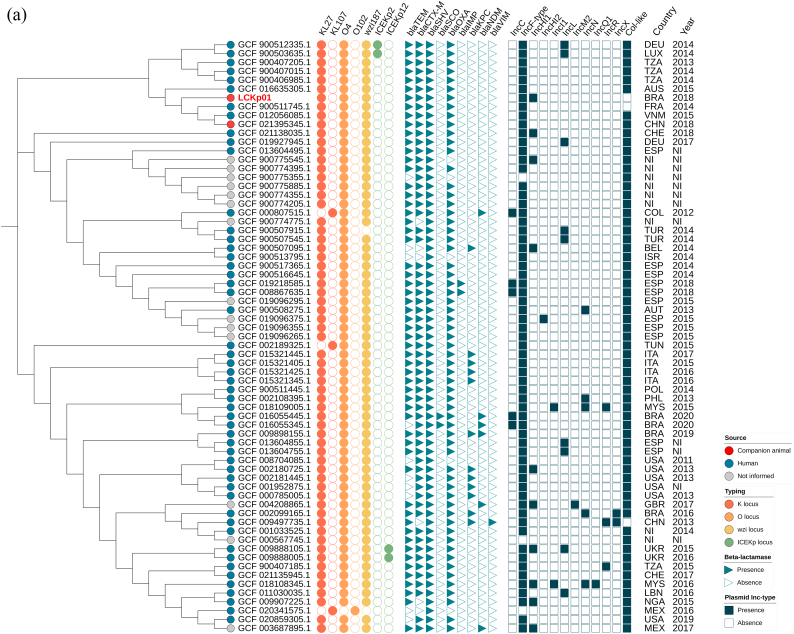

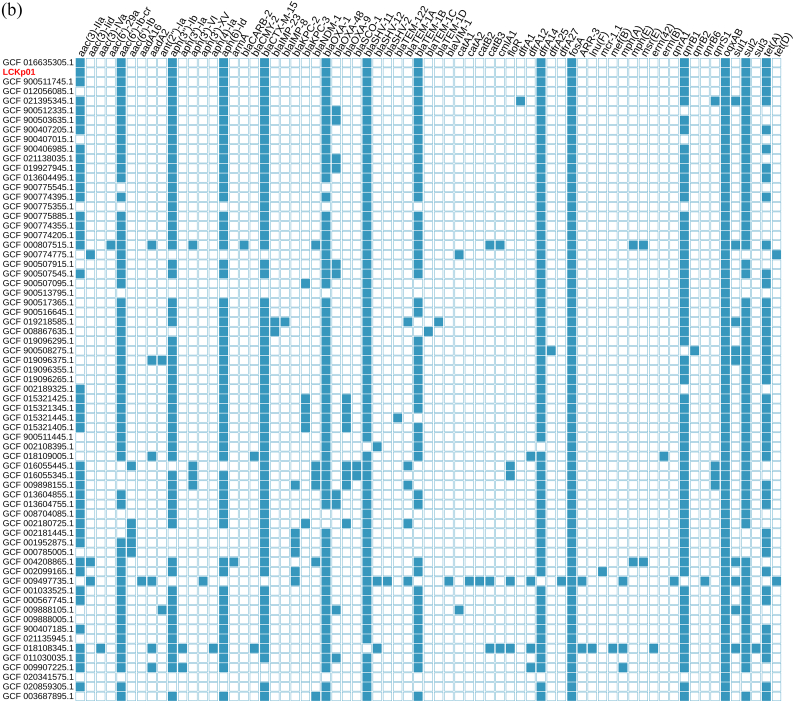
In A, Single nucleotide polymorphism (SNP)-based phylogenomic relationship of 67 *K. pneumoniae* ST392 strains isolated globally from human and animal sources. The LcKp01 strain clustered (60–80 SNPs differences) with four *Klebsiella pneumoniae* ST392 strains isolated from human and animal samples, in Australia, France, Vietnam and China. Heatmap includes all 67 *Klebsiella pneumoniae* ST392 capsular serotyping, capsule-associated virulence genes, ESBL/carbapenemase genes, plasmid replicons, and epidemiological information (country and year). The companion animal strain analyzed in this study (accession number: JAEDYP000000000) is represented by red color. NI, Not informed. Countries are labeled according to ISO 3166-1 Alpha-3 code, as follows: AUS, Australia; BRA, Brazil; FRA, France; VNM, Vietnam; CHN, China; DEU, Germany; LUX, Luxembourg; TZA, Tanzania; CHE, Switzerland; ESP, Spain; COL, Colombia; TUR, Turkey; BEL, Belgium; ISR, Israel; AUT, Austria; TUN, Tunisia; ITA, Italy; POL, Poland; PHL, Philippines; MYS, Malaysia; USA, United States of America; GBR, United Kingdom; UKR, Ukraine; LBN, Lebanon; NGA, Nigeria; MEX, Mexico. Tree topology and scale bar were automatically generated in scale by default parameters of iTol v.6 and refers to branch lengths, which are measured in number of substitutions per site. In B, Heatmap displaying the acquired antibiotic resistance genes identified in all 67 *K. pneumoniae* ST392 genomes analyzed in this study. Light blue and white filled squares indicate gene presence and absence, respectively. (For interpretation of the references to color in this figure legend, the reader is referred to the web version of this article.)

SNP-based phylogenomic analysis revealed SNPs differences (0 to 251) among all 67 *K. pneumoniae* ST392 genomes. The LCKp01 strain clustered with four strains (60–80 SNPs differences) isolated from humans (Australia, 2015; France, 2014; Vietnam, 2015) and animal (China, 2018) samples (GenBank accession numbers: WMMT00000000.1, UKID00000000.1, BHWE00000000.1 and JAJVRO000000000.1, respectively) (Fig. 2A and Supplementary Table S1).

Resistome analysis revealed the presence of genes conferring resistance to β-lactams (*bla*_CTX-M-15_, *bla*_SHV-11_, *bla*_OXA-1_, and *bla*_TEM-1D_), aminoglycoside [*aac(6′)-Ib-cr*,*aac(3)-lla*, *strA*, and *strB*], tetracycline (*tetA*), trimethoprim (*dfrA14*), sulphonamides (*sul2*), and fosfomycin (*fosA*). Moreover, chromosomal point mutations in the quinolone resistance-determining region (QRDR) of *gyrA* (S83I) and *parC* (S80I), and detection of plasmid-mediated quinolone resistance (PMQR) genes (*oqxA, oqxB*, and *qnrB1*) were associated with fluoroquinolone resistance (Fig. 2B). Additionally, KL27 (wzi-187) and O-locus 4 (O4), which encode the polysaccharide capsule and the lipopolysaccharide O antigen, respectively, were also detected (Fig. 2A).

While plasmid replicons IncHI1B, IncFIB, and IncFII were identified in the *K. pneumoniae* LCKp01 strain, plasmidome analysis showed that Col-like and IncF-type replicons have been the most frequent plasmids carried by *K. pneumoniae* strains belonging to ST392, followed by IncHI1, IncL, IncC, IncN, IncR, IncI1, IncX, IncHI2, IncM2, and IncQ1 replicons (Fig. 2A and Supplementary Table 1). On the other hand, *K. pneumoniae* ST392 producing CTX-M-15 seem to carry various IncF-type plasmid multilocus sequence types (*i.e.,* IncF[K2:A-:B-], IncF[K12:A-:B-], IncF[K7:A-:B-], IncF[K7:A-:B36], and IncF[F-:A13:B-]), with K7:A-:B- exhibiting the highest prevalence (54/67; 80,59%). Interestingly, pMLST K7:A-:B- plasmids have been previously described among CTX-M-15-producing *K. pneumoniae* ST983 strains isolated from hospitalized patients in Malaysia [16], Tanzania [17], and South Africa [18].

In summary, we report genomic data of *K. pneumoniae* ST392 harboring *bla*_CTX-M-15_ and other clinically important antimicrobial resistance genes, isolated from an infected companion animal in South America. Currently, WGS-based studies on ESBL-positive *K. pneumoniae* from companion animals remain scarce [19]. Additionally, phylogenomic information on human-associated clones of *K. pneumoniae* recovered from dogs and cats is also poorly investigated [19]. Considering that *K. pneumoniae* ST392 has been so far predominantly reported in human patients, our findings suggest that this *K. pneumoniae* clone may spread beyond human hospital settings and affecting now hospitalized pets. Last but not least, our data could be helpful for comparative genomic analyses of *K. pneumoniae* ST392 strains that could emerge at the human-animal-environment interface, since our early phylogeographical analysis confirmed successful expansion of ST392 as a global clone of One Health concern.

The following is the supplementary data related to this article.

Supplementary Table S1 SNP-based matrix of *Klebsiella pneumoniae* ST392 genomes. Supplementary Table S1

## Data availability

The data that support the findings of this study are available from the corresponding author upon reasonable request. This Whole Genome Shotgun project has been deposited at DDBJ/ENA/GenBank under the accession JAEDYP000000000**.** The version described in this paper is version one. In addition, genomic information of *K. pneumoniae* LCKp01 strain is available on the OneBR platform under the number ID ONE207 (http://onehealthbr.com/).

## Ethics statement

The authors confirm that the ethical policies of the journal, as noted on the journal's author guidelines page, have been adhered to. No ethical approval was required for this specific study.

## Funding statement

This study was supported by the 10.13039/100000865Bill and Melinda Gates Foundation (Grand Challenges Explorations Brazil OPP1193112). Under the grant conditions of the Foundation, a CC BY or equivalent license is applied to the Author Accepted Manuscript version arising from this submission. Additionally, this study was supported by the 10.13039/501100001807Fundação de Amparo à Pesquisa do Estado de São Paulo (2020/08224-9, 2019/15778-4), 10.13039/501100003593Conselho Nacional de Desenvolvimento Científico e Tecnológico (AMR 443819/2018-1, 312249/2017-9, 422984/2021-3 and 314336/2021-4), and 10.13039/501100002322Coordenação de Aperfeiçoamento de Pessoal de Nível Superior (88882.333054/2019-01). NL is a research fellow of 10.13039/501100003593CNPq (314336/2021-4). BC and HF were researcher fellows of 10.13039/501100002322CAPES (88882.333054/2019-01; 88887.506496/2020-00). FE was a research fellow of 10.13039/501100001807FAPESP (2019/15578-4).

## CRediT authorship contribution statement

**Luciano C.B.A. da Silva:** Conceptualization, Methodology, Investigation, Writing – original draft. **Brenda Cardoso:** Conceptualization, Methodology, Data curation, Writing – original draft, Writing – review & editing. **Herrison Fontana:** Methodology, Investigation, Data curation, Writing – original draft. **Fernanda Esposito:** Methodology, Investigation, Data curation, Writing – review & editing. **Silvia R. Cortopassi:** Investigation, Writing – review & editing. **Fábio P. Sellera:** Conceptualization, Methodology, Investigation, Writing – original draft, Writing – review & editing. **Nilton Lincopan:** Conceptualization, Resources, Writing – review & editing, Funding acquisition.

## Declaration of Competing Interest

All authors declare no conflicts of interest.
